# The obesity paradox in Japanese COVID-19 patients

**DOI:** 10.7150/ijms.86933

**Published:** 2023-09-11

**Authors:** Junichi Yoshida, Kaoru Setoguchi, Kenichiro Shiraishi, Tetsuya Kikuchi, Masao Tanaka

**Affiliations:** 1 Infection Control Committee, Shimonoseki City Hospital, Shimonoseki, Japan.; 2 Department of Anesthesiology, Shimonoseki City Hospital, Shimonoseki, Japan.; 3 Department of Medicine and Biosystemic Science, Kyushu University Graduate School of Medical Sciences, Fukuoka, Japan.

**Keywords:** risk, SARS-CoV-2, high flow nasal cannula oxygenation, chemotherapy

## Abstract

**Purpose:** To investigate the effect of obesity on mortality and invasive respiratory care (IRC) in patients with COVID-19.

**Methods:** We studied 1,105 patients for 34 months and collected data. The primary outcome was all-cause death at 29 days. The secondary outcome was IRC indicated by a pulse oximetry rate below 93% at a mask oxygenation rate of 5 L/min or more.

**Results:** Age- and sex-adjusted multivariate regression analysis for 29-day deaths showed the significance of body mass index (BMI) > 19.6 kg/m^2^ (odds ratio 0.117, 95% confidence interval 0.052-0.265, *P*<0.001). The graphs with BMI in the abscissa showed, within a BMI between 11 and 25 kg/m^2^, a decreasing pattern for mortality and IRC rate, and no increase in overweight.

**Conclusion:** In Japanese COVID-19 patients, the risk of mortality and the IRC rate decreased in underweight patients and remained low in overweight patients, suggesting the importance of the obesity paradox.

## Introduction

The “obesity paradox” has been described as the paradoxical decrease in morbidity and mortality in overweight patients compared to patients that are underweight 1. Among patients with COVID-19, the same pattern has been observed 2. Watanabe et al 3 reported that in older Japanese adults, a U-shaped relationship with body mass index (BMI) in the abscissa is observed. This phenomenon has also been reported in the treatment of COVID-19, including a J-shaped relationship 4. Likewise, Manolis et al. 5 depicted a J-curve pattern of the relationship between BMI and the risk of COVID-19 infection. However, other studies have refuted this claim 1,6. Therefore, in this study, we evaluated the effect of BMI on the outcomes of patients with COVID-19.

## Material and methods

### Subjects

We included all inpatients admitted between March 3, 2020, and December 31, 2022, and collected data on death, demographics, the body mass index (BMI), the body surface area (BSA), the use of remdesivir, SARS-CoV-2 vaccination, concurrent chemotherapy, and laboratory results. Demographic data included nationality in terms of international differences 7. The study period was divided into two eras due to differences in mortality and vaccination status. The laboratory data included neutrophil to lymphocyte ratio (NLR).

### Methods

The primary outcome was all-cause death at 29 days. During the COVID-19 pandemic, Hancı et al. 8 reported the success of high-flow nasal cannula oxygenation as one of intensive respiratory care (IRC). For IRC, we defined the inclusion criterion as a pulse oximetry rate below 93% at a mask oxygenation rate of 5 L/min or more.

The two outcomes were first calculated as crude rates, which were adjusted for age and sex. SPSS (version 28; IBM Inc., Armonk, NY, USA) was used for the statistical analysis. Continuous variables underwent receiver operating characteristics analyses to determine cut off values. Using these results, we performed multivariate analyses to see independent risk factors for the outcomes of 29-day mortality or IRC. Statistical significance was determined at *P* < 0.05.

### Ethics Committee Approval and Patient Consent

The authors obtained ethical approval to conduct the study and permission to use the data from the Internal Review Board of the institute with permission code 2023SCHEC-037. Thereby, the need for informed consent was waived in accordance with the Chapter 5, Part 12, B Research not involving invasiveness, (b) Research not involving intervention of the Ethical Guideline from the Ministry of Education, Culture, Sports, Science and Technology, Japan.

## Results

Of the 1,105 patients, males accounted for 554 (50.1%) and Japanese citizens accounted for 1,097 (99.3%). Death by day 29 was observed in 32 patients (2.9%), including the diagnoses of COVID-19 per se (N = 20), bacterial pneumonia (N = 7), septic shock (N = 1), and urinary tract infection (N = 1) among infectious diseases. The remaining was cardiovascular events (N = 3). None of the patients (N = 8) with BMI > 19.6 kg/m^2^ died of bacterial pneumonia. In 29.2% (7/24) patients with BMI<= 19.6 kg/m^2^. however, bacterial pneumonia led to deaths. IRC was observed in 37 patients (3.3%).

**Figure 1:**
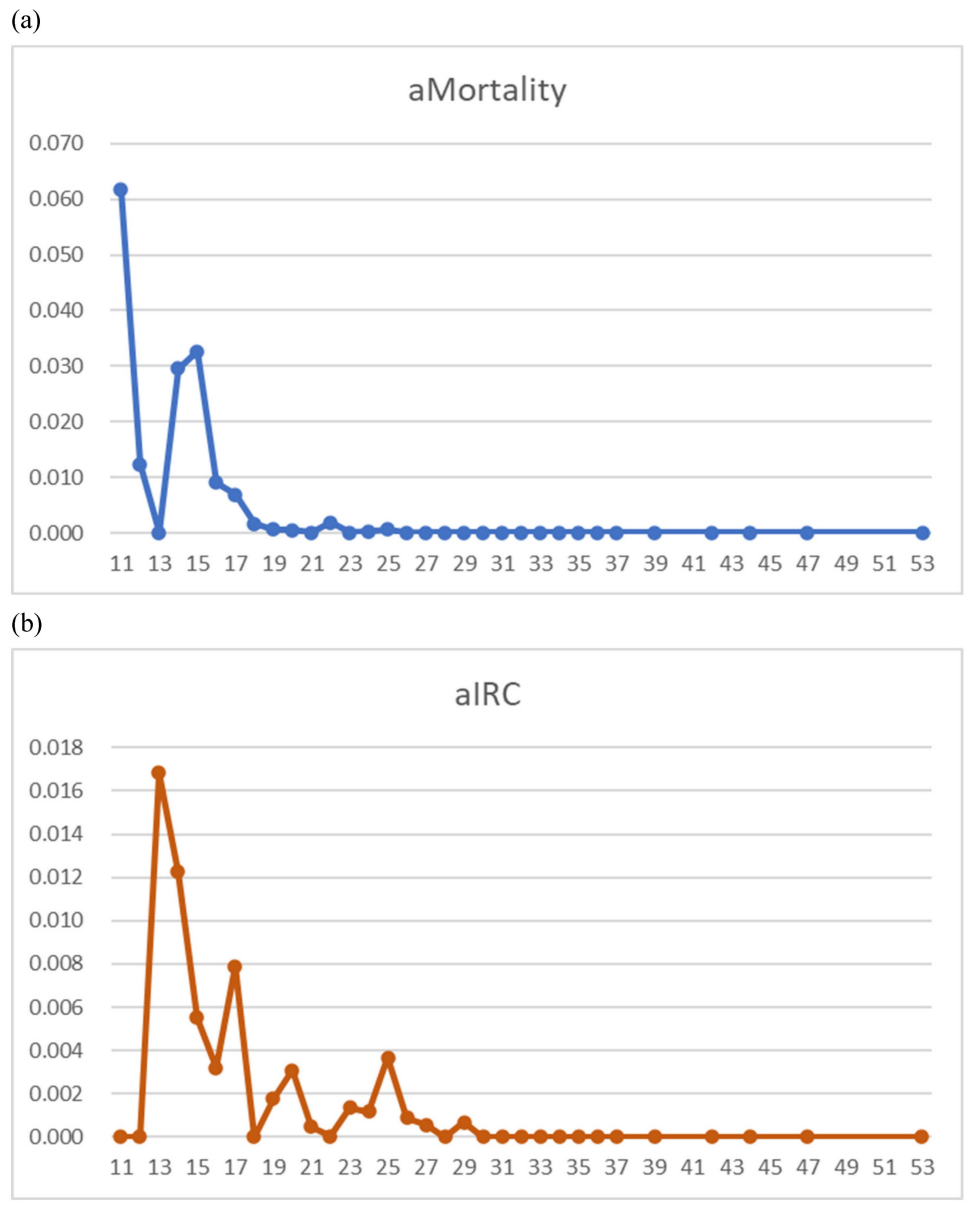
Age- and sex-adjusted 29-day mortality (aMortality) and intensive respiratory care rate (aIRC) illustrated by body mass index (BMI, x-axis) in COVID-19 patients (N=1,105). (1-a) aMortality demonstrates peaks at BMIs of 11-15 and 22 kg/m^2^. (1-b) aIRC shows a decreasing trend.

**Figure 2:**
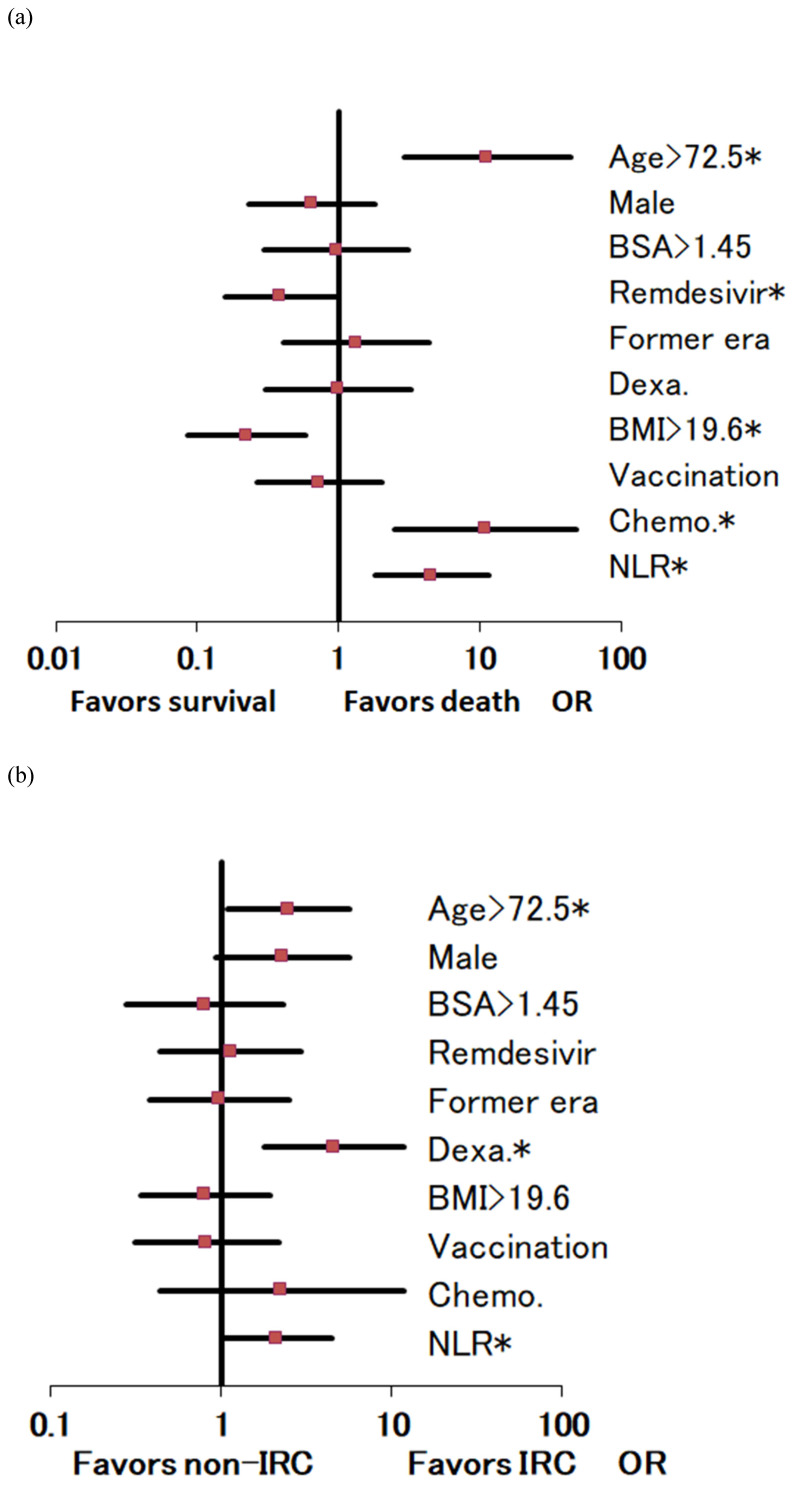
Odds ratio (OR) and 95% confidence interval of risk factors by multivariate regression analyses. (2-a) For 29-day mortality, significant risks were high age, concurrent chemotherapy (Chemo.), and high neutrophil to lymphocyte ratios (NLR). However, high levels of body mass index (BMI) and remdesivir use favored survival. (1-b) For invasive respiratory care (IRC), significant risks were high age, dexamethasone use (Dexa.), and high NLR. Note: BSA, body surface area; *, statistical significance (*P*<0.05).

Age- and sex-adjusted 29-day mortality by BMI demonstrated peaks at BMIs of 11-15 and 22 kg/m^2^ (Figure 1-a). Age- and sex-adjusted multivariate regression analysis for 29-day deaths showed the significance in BMI > 19.6 kg/m^2^ (odds ratio 0.117, 95% confidence interval 0.052-0.265, *P* < 0.001) and in the remdesivir use (*P* = 0.040), both favoring survival (Figure 2-a). On the contrary, significant mortality risks were age > 72.5 (*P* < 0.001), concurrent chemotherapy (*P* = 0.001), and NLR levels > 3.65 (*P* = 0.001) (Figure 2-a). The total numbers of the deaths / chemotherapy were 3/24, respectively, and their subtotals included hematologic malignancy (1/3), thoracic cancers (2/7), and others (0/14).

For IRC, significant risks were age > 72.5 (*P* = 0.031), dexamethasone use (*P* = 0.002), and NLR levels > 3.65 (*P* = 0.048) (Figure 2-b). The graphs with BMI in the abscissa showed for a BMI between 11 and 25 kg/m^2^, a decreasing pattern for IRC rate and no increase in overweight (Figure 1b).

## Discussion

Our study on COVID-19 mortality indicated that BMI presented a negative risk, reflecting the low BMI in Japanese patients 7. Thus, the “obesity paradox” in COVID-19 mortality may take the form of low BMI at risk. In Korea, Kang and Kong 9 reported that COVID-19 patients with a BMI of < 18.5 kg/m^2^ and those with a BMI ≥ 25 kg/m^2^ had a high risk of fatality in Korea. Similarly, Singh et al. 10 reported that being underweight (BMI < 18 kg/m^2^) was associated with an increased risk of mortality. Similarly, Jung et al. 11 adjusted for age and sex to assess the effects of BMI.

Kananen et al. 12 described the role of being underweight and malnourished in the in-hospital mortality of patients with COVID-19. Bouziotis and Preiser 13 reported that the mortality rate in underweight patients was higher than that in overweight patients. Thus, the risk of being underweight may exceed that of being obese in patients with COVID-19. None of the patients with BMI > 19.6 kg/m^2^ died of bacterial pneumonia whereas 29.2% of those with BMI <= 19.6 kg/m^2^ died of this disease. This contrast in patients with BMI > 19.6 kg/m^2^ and with BMI <= 19.6 kg/m^2^ may have contributed to the U-shape on the graph of BMI vs. mortality, the former being from SARS-CoV-2 viral cause and the latter from bacterial cause.

Regarding cancer treatment, Curley et al. 14 reported that COVID-19 mortality was affected by age, sex, and comorbidities. Our results, adjusted for age and sex, showed that ongoing chemotherapy had a high odds ratio for 29-day mortality. The breakdowns of the chemotherapy showed that most of the dead patients carried hematologic malignancy or thoracic cancers. Poor prognosis carried by these neoplasms and/or adverse events inherent to the chemotherapy may have contributed to 29-day mortality, although no malignant direct diagnoses were documented in the dead patients.

Coss-Rovirosa et al. 15 reported that the risk presented by invasive mechanical ventilation in COVID-19 patients increases in patients with a BMI > 35 kg/m^2^. The World Health Organization 16 reported a BMI of < 18.5 kg/m^2^ as underweight in the Asian population, which corresponds to our decreasing intervals for mortality and IRC rates. Thus, underweight Japanese nationals may exhibit a decreasing linear pattern.

Our multivariate analysis for IRC, however, failed to show significance in BMI, but demonstrated significance in high age, dexamethasone use, and high NLR. As Hol et al. 17 reported, higher age was associated with more complications, longer length of stay in hospital and a higher mortality. Indeed, the 29-day mortality in our study showed significant risk in high age. The reason for dexamethasone at independent risk of IRC was treating cytokine storm in patients with IRC. Likewise, Wagner and others 18 stated that systemic corticosteroids are used to treat people with COVID-19 because they counter hyper-inflammation. Thus, the dexamethasone use may have reflected its coexistence with IRC, not being at risk of IRC.

As regards NLR and COVID-19, La Torre and others 19 reported that a high NLR suggests worse survival. The authors described that NLR provides an indirect index of the patient's inflammatory state to predict worsening of chronic diseases such as respiratory, cardiovascular and renal conditions 19. Likewise, our study revealed high NLR at independent risk of IRC and of 29-day mortality. However, further evaluation for the mechanisms of NLR at risk of IRC and mortality was prevented by missing values to perform logistic regression analyses.

As a limitation of our study, the hip-to-waist ratio may better represent obesity than BMI. Serpa Neto et al. 20 reported that BSA in addition to BMI predicted well in-hospital mortality in critically ill patients under invasive mechanical ventilation. BSA levels in our study, however, failed to show significance in the multivariate analyses in mortality and IRC. It remains undetermined whether or not the discrepancy was due to racial difference between the Japanese and the Occidentals.

Another limitation may involve the short term of 29 days for mortality. This term was based on the clinical trial of remdesivir 21, which showed decreased 29-day mortality using remdesivir as in our multivariate analysis. In future, studies with longer follow up are awaited for mortality analyses.

## Conclusion

In Japanese COVID-19 patients, the risk of mortality and the IRC rate decreased in underweight patients and remained low in overweight patients, suggesting the importance of the obesity paradox.

## Author contributions

JY and KS (Setoguchi) contributed substantially to the conception or design of the work, the acquisition, analysis, or interpretation of data for the work. JY and KS (Shiraishi) performed drafting the work or reviewing it critically for important intellectual content. All the authors approved the final version to be published. All the authors gave agreement to be accountable for all aspects of the work in ensuring that questions related to the accuracy or integrity of any part of the work was appropriately investigated and resolved.

## Abbreviations

BMI: body mass index
BSA: body surface area
NLR: neutrophil to lymphocyte ratio
IRC: intensive respiratory care
